# MicroRNA-146a suppresses ROCK1 allowing hyperphosphorylation of tau in Alzheimer’s disease

**DOI:** 10.1038/srep26697

**Published:** 2016-05-25

**Authors:** Gang Wang, Yue Huang, Li-Ling Wang, Yong-Fang Zhang, Jing Xu, Yi Zhou, Guinevere F. Lourenco, Bei Zhang, Ying Wang, Ru-Jing Ren, Glenda M. Halliday, Sheng-Di Chen

**Affiliations:** 1 Department of Neurology & Neuroscience Institute, Ruijin Hospital affiliated to Shanghai Jiao Tong University School of Medicine, Shanghai, 200025, China; 2 Neuroscience Research Australia & University of New South Wales, Randwick, 2031, New South Wales, Australia; 3 Research Laboratory of Cell Regulation, School of Medicine, Shanghai Jiaotong University, 280 South Chongqing Road, Shanghai 200025, China; 4 Laboratory of Neurodegenerative Diseases, Institute of Health Science, Shanghai Institutes of Biological Sciences, Chinese Academy of Sciences and Shanghai Jiao Tong University School of Medicine, Shanghai 200025, China

## Abstract

MicroRNA-146a is upregulated in the brains of patients with Alzheimer’s disease (AD). Here, we show that the rho-associated, coiled-coil containing protein kinase 1 (ROCK1) is a target of microRNA-146a in neural cells. Knockdown of ROCK1 mimicked the effects of microRNA-146a overexpression and induced abnormal tau phosphorylation, which was associated with inhibition of phosphorylation of the phosphatase and tensin homolog (PTEN). The ROCK1/PTEN pathway has been implicated in the neuronal hyperphosphorylation of tau that occurs in AD. To determine the function of ROCK1 in AD, brain tissue from 17 donors with low, intermediate or high probability of AD pathology were obtained and analyzed. Data showed that ROCK1 protein levels were reduced and ROCK1 colocalised with hyperphosphorylated tau in early neurofibrillary tangles. Intra-hippocampal delivery of a microRNA-146a specific inhibitor (antagomir) into 5xFAD mice showed enhanced hippocampal levels of ROCK1 protein and repressed tau hyperphosphorylation, partly restoring memory function in the 5xFAD mice. Our *in vitro* and *in vivo* results confirm that dysregulation of microRNA-146a biogenesis contributes to tau hyperphosphorylation and AD pathogenesis, and inhibition of this microRNA could be a viable novel *in vivo* therapy for AD.

Alzheimer’s disease (AD) is a neurodegenerative disorder characterized by progressive memory loss and increasing dysfunction in mental behavior. Diagnostic neuropathological features including extracellular amyloid plaques consisting of deposits of beta-amyloid (Aβ), intracellular neurofibrillary tangles consisting of hyperphosphorylated tau protein, and neuronal cell loss^1^. In fact, the density and distribution of neurofibrillary tangles in the brain correlates with the severity of dementia^2^, with the tau protein abnormally phosphorylated in the brains of AD patients. Under normal circumstances, tau binds to microtubules stabilizing the axonal cytoskeleton^3^. However, hyperphosphorylation of tau decreases its ability to bind to microtubules, and consequently leads to microtubule destabilization, disruption of the axonal transport system, and ultimately, the formation of neurofibrillary tangles and neuronal loss^4^,^5^,^6^,^7^,^8^,^9^,^10^. Previous studies have shown that increasing tau phosphorylation occurs early in the development of AD^11^,^12^, and that Aβ associated clinical decline is thought to occur only following such elevated tau phosphorylation^11^,^13^. Therefore, defining the upstream regulation of tau hyperphosphorylation might lead to the identification of novel targets for AD.

MicroRNAs are short, conserved, non-coding RNAs that negatively regulate gene expression post-transcriptionally by binding to the 3′ untranslated regions (3′ UTR) of their target mRNA^14^. Because of their size, multiple microRNAs can suppress the expression of a single gene simultaneously, and likewise a single microRNA can have several or even hundreds of target genes and influence multiple pathways at the same time^14^. As microRNA expression patterns are tissue-specific, their dysregulation has been identified in a large number of human diseases^14^.

In AD, a number of dysregulated microRNAs have been identified but one stands out due to its levels early in disease and in diverse biological samples, as well as its central role in multiple pathways involved in AD^15^ – the microRNA-146a is perhaps best known for its role in the innate immune response^16^, although it is abundant in both mouse and human brain^17^ and expressed in both microglia and importantly neurons^18^. Upregulation of microRNA-146a occurs in preclinical AD in serum^19^ and in the hippocampus, the brain region where the neurons are most affected early by AD^20^, as well as at diagnosis in cerebrospinal fluid^21^ and affected brain regions^21^,^22^,^23^, but its levels are reduced by end stage disease^19^,^20^,^24^ when neuronal loss and tissue damage is marked. Recent bioinformatics analysis of microRNA pathways in AD identified microRNA-146a as a central player in eight of the nine active regulatory pathways underlying this disease^15^.

As microRNA-146a is upregulated early in the hippocampus, we wondered if it had a role in the most marked pathology in the hippocampus, that is the hyperphosphorylation of tau (see above). We therefore sought to test whether any protein targets of microRNA-146a could influence tau phosphorylation. In peripheral cell systems, microRNA-146a suppresses the gene rho-associated, coiled-coil containing protein kinase 1 (ROCK1)^25^,^26^ and ROCK1 binding to the protein phosphatase and tensin homolog (PTEN) is an essential step for PTEN phosphorylation which promotes tau dephosphorylation^27^,^28^,^29^,^30^. Importantly, a decrease in the phosphorylation of PTEN and PTEN immunoreactive temporal lobe pyramidal neurons is observed in AD^31^. This data suggests that microRNA-146a upregulation might contribute to abnormal tau hyperphosphorylation in neurons by regulating the ROCK1-PTEN signaling pathway in neurons. In systematically testing for evidence of this, we show that ROCK1 is a target of microRNA-146a in neural cells, that overexpression of microRNA-146a in neural cells induces tau hyperphosphorylation via ROCK1 regulation through PTEN, that there is a reduction in ROCK1 levels and a colocalisation with hyperphosphorylated tau in neurofibrillary tangles in the brains of patients with AD, and that inhibiting microRNA-146a in a validated mouse model of AD reduces tau hyperphosphorylation and enhances memory function. The findings of these experiments support the proposition that microRNA-146a plays an important role in the pathophysiology of AD by regulating tau phosphorylation.

## Results and Discussion

### ROCK1 is a target of microRNA-146a in neural cells

**Figure 1:**
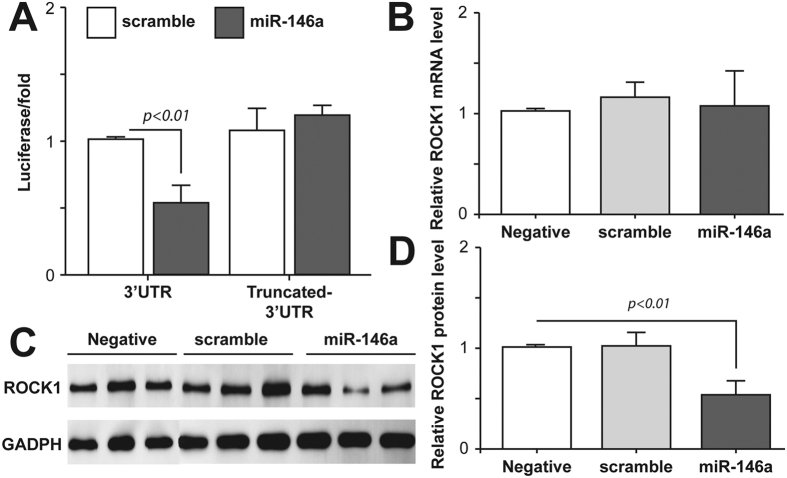
ROCK1 is a target of microRNA-146a in neural cells. (**A**) The effect of microRNA-146a (miR-146a) on ROCK1 expression was assessed in neural SH-SY5Y cells using a luciferase reporter system. A vector expressing miR-146a and a psiCHECK-2 vector containing either the full length ROCK1 3′ UTR or a truncated 3′ UTR lacking the miR-146a binding sites were co-transfected into neural SH-SY5Y cells. In control cells the vector expressing miR-146a was replaced with a scrambled miR-146a vector (scramble). Fluorescence was measured 48 hours after transfection. MiR-146a bound to the full length ROCK1 3′ UTR and inhibited its activity, but did not bind to the truncated ROCK1 3′ UTR. **(B)** Real-time PCR was used to detect the mRNA expression level of ROCK1 in neural SH-SY5Y cells. Overexpression of miR-146a or scramble did not affect the relative ROCK1 mRNA level compared to neural cells without vectors (negative). Relative expression of ROCK1 mRNA was calculated using the ΔΔCT method. **(C)** Western blot was used to measure ROCK1 protein translation in neural cells. Overexpression of miR-146a significantly decreased expression of endogenous ROCK1 protein in neural SH-SY5Y cells compared to negative or scramble treated neural cells. **(D)** Overexpression of miR-146a significantly reduced the relative ROCK1 protein level compared to negative or scramble treated neural cells. All of the data are expressed as means ± SD of at least three independent experiments.

ROCK1 has been identified as a target of microRNA-146a in peripheral cells^25^,^26^ but whether it has a similar target in neurons is not known. The ROCK1 3′ UTR contains three complementary sites to the seed region of microRNA-146a (miRTarBase) located at nucleotides 310–331, 774–798, and 1008–1029 in the ROCK1 transcript (Supplementary Fig. 1, blocked and underlined). Co-transfection of neural SH-SY5Y cells with a luciferase reporter plasmid containing the 3′ UTR of ROCK1 and a microRNA-146a expression vector showed that the expression of microRNA-146a significantly repressed the luciferase activity of reporter construct containing the ROCK1 3′ UTR in these neural cells (Fig. 1A). When a control plasmid containing scrambled microRNA-146a was used, both luciferase and microRNA-146a expression were not affected. To further validate that microRNA-146a was able to bind to the ROCK1 3′ UTR in neural cells, we amplified a truncated form of the ROCK1 3′ UTR that lacked the binding sites for microRNA-146a and cloned it into a luciferase reporter vector. Expression of microRNA-146a and the truncated ROCK1 3′ UTR had no effect on luciferase activity in neural cells (Fig. 1A). Overexpression of microRNA-146a did not affect the level of endogenous ROCK1 mRNA expression in the neural SH-SY5Y cells (Fig. 1B). However, as shown in Fig. 1C,D, overexpression of microRNA-146a significantly repressed the translation of endogenous ROCK1 protein in these neural cells. Our results suggest that microRNA-146a exerts posttranscriptional repression on ROCK1 by inhibition of protein translation via binding to the ROCK1 mRNA 3′ UTR rather than mRNA cleavage.

### MicroRNA-146a overexpression in neural cells induces tau hyperphosphorylation via ROCK1 regulation through PTEN

**Figure 2:**
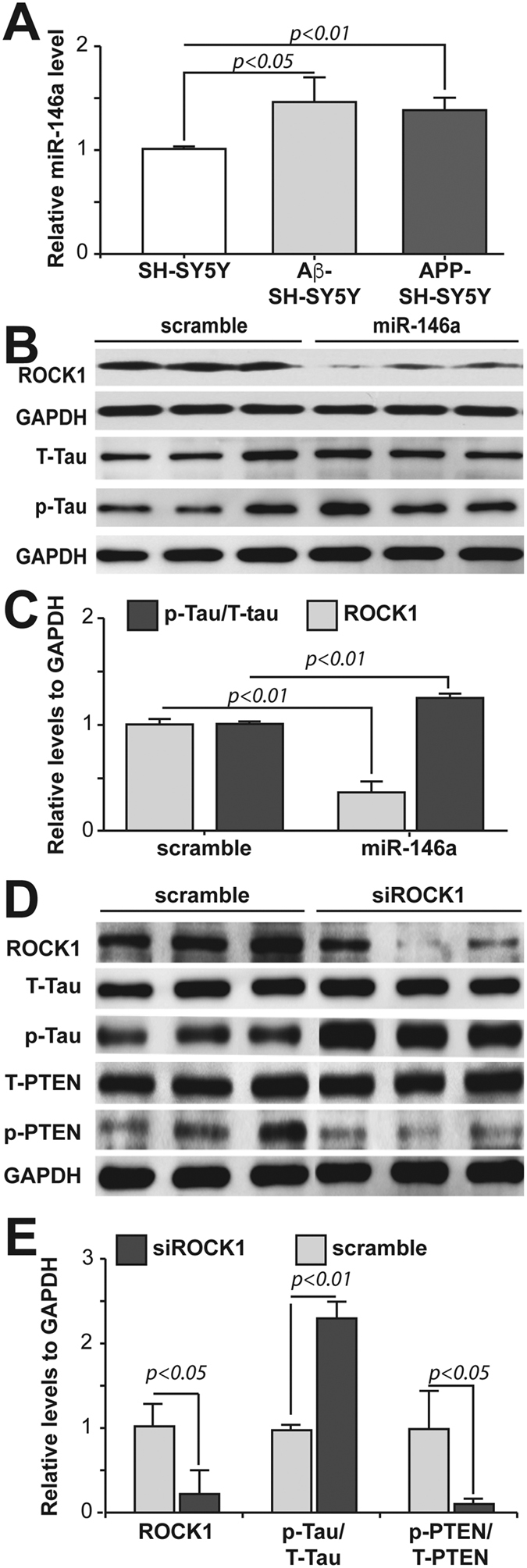
Molecular mechanisms of microRNA-146a associated with Alzheimer pathologies. (**A**) Conditions inducing microRNA-146a (miR-146a) overexpression in neural SH-SY5Y cells included treatment with Aβ_1–42_ (5 μM) for 24 hours and stable overexpression of the APP Swedish mutation compared to untransfected control neural cells (means ± SD). **(B,C**) Overexpression of miR-146ain neural SH-SY5Y cells induces decreased levels of ROCK1 and tau hyperphosphorylation, as demonstrated in Western blots of total tau (T-Tau) and phosphorylated tau (Ser396, p-Tau) (means ± SD). Control neural cells expressed a scrambled miR-146a vector (scramble). **(D,E)** Knockdown of ROCK1 mRNA using siRNA in neural SH-SY5Y cells caused an increase in tau phosphorylation at Ser396 (p-Tau) compared to total tau (T-Tau) and decreased PTEN phosphorylation (p-PTEN) compared to total PTEN (T-PTEN) as detected in Western blots using an antibody specific for phosphorylation at Ser380/Thr382/Thr383. Scrambled ROCK1 siRNA (scramble) was used in control neural cell experiments. Quantified data are expressed as mean ± SD for at least three independent experiments.

Given that ROCK1 mRNA is a target of microRNA-146a regulation in neural cells, we next determined that microRNA-146a was upregulated in two neural cell models of AD (Fig. 2A). Previous studies had shown that in mixed co-cultures of neurons and glia, treatment with Aβ_1–42_ slightly increases microRNA-146a^22^,^32^, as shown in our single culture system. These previous studies emphasized the downstream inflammatory pathways involved in the co-culture system^22^,^32^, while our data in pure neural cultures shows that additional neural pathways are also involved and should not be ignored.

We next assessed whether increasing microRNA-146a affected tau phosphorylation and the cytoskeleton in neural cells, as tau hyperphosphorylation and microtubule destabilization is widely acknowledged in AD^33^,^34^,^35^. Overexpression of microRNA-146a in the neural SH-SY5Y cells significantly increased tau phosphorylation, while simultaneously inhibiting ROCK1 protein translation (Fig. 2B,C). Evident microtubule cytoskeleton disruption was observed in the neural SH-SY5Y cells overexpressing microRNA-146a compared to the control cells (data not shown). To our knowledge, this is the first report of a novel inhibitory interaction between microRNA-146a and the ROCK1 3′ UTR in neural cells that contributes to phospho-tau regulation. This is consistent with the previous studies in AD brain tissue showing microRNA-146a is selectively upregulated in brain regions most affected by tau pathology, such as the hippocampus and temporal cortex^20^,^21^,^22^,^23^.

ROCK1 protein has been shown to bind to and phosphorylate the tumor suppressor PTEN^29^,^30^,^36^. It is well established that the phosphatase PTEN dephosphorylates tau, and conversely, that a loss of PTEN function can cause neurodegeneration mediated by hyperphosphorylation of tau and neurofibrillary tangle formation^27^,^28^,^37^. Since overexpression of microRNA-146a significantly decreased ROCK1 protein translation and significantly increased tau phosphorylation, we next investigated whether PTEN phosphorylation was involved in this process using siRNA to knockdown ROCK1 in the neural SH-SY5Y cells. The transfection efficiency was assessed by counting the percentage of fluorescein–labelled cells using fluorescence microscopy, and was approximately 80%. The level of ROCK1 protein was significantly decreased after transfection with ROCK1 siRNA but not scramble control siRNA (Fig. 2D). Decreased ROCK1 protein levels reduced PTEN phosphorylation at Ser380/Thr382/Thr383, and increased tau phosphorylation at Ser396 (Fig. 2E). Importantly, in brain regions with hyperphosphorylated tau in patients with AD there is a reduction in the phosphorylation but not the amount of PTEN^31^. Reduced phosphorylation of PTEN can dramatically increase tau phosphorylation and impair the ability of tau to bind to microtubules^27^. Our data knocking down ROCK1 protein translation caused a reduction in PTEN phosphorylation and induced tau hyperphosphorylation, indicating that this is a plausible reason for the change in PTEN phosphorylation in AD. However, we could not exclude there exists another kinase which also interacts with ROCK1 and is involved in hyperphosphorylation of tau.

### ROCK1 is reduced inpatients with Alzheimer’s disease (AD) and colocalises with phospho-tau-immunoreactive tangles

**Figure 3:**
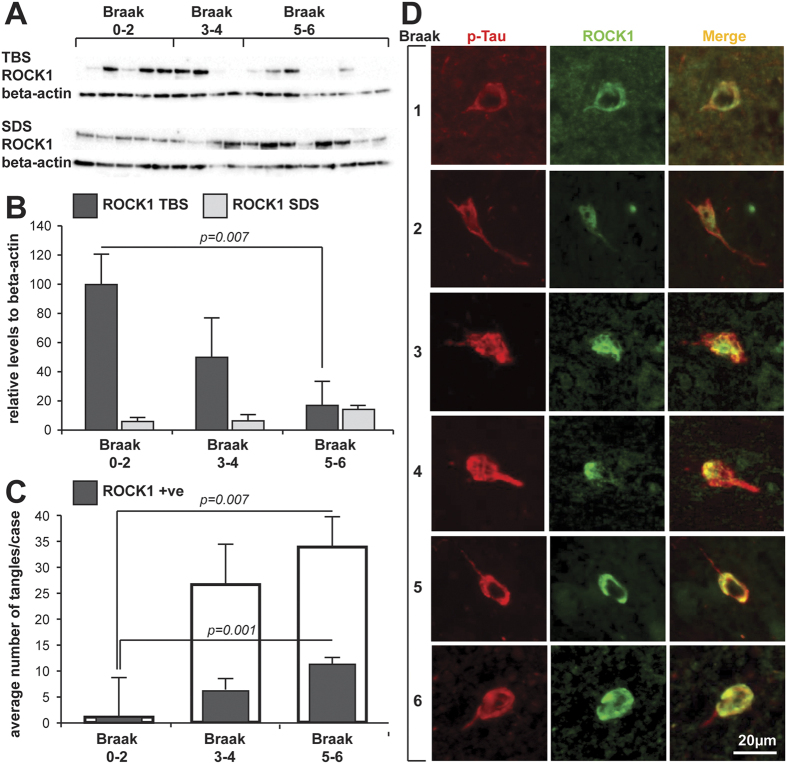
ROCK1 in human brain tissue. (**A**) Western blot of ROCK1 protein levels in the different tissue fractions (TBS = soluble fraction, SDS = SDS fraction, see methods) of the inferior temporal lobe of donors at different stages of AD pathology. (**B**) Relative quantitation of the ROCK1 Western blots in the different tissue fractions shown in (**A**). (**C**) Quantitation of the numbers of phospho-tau-immunoreactive tangles (open bars) and those colocalising ROCK1 (shaded bar) at different stages of AD. Significant increases in both types of tangles occur with increasing stages of AD. (**D**) Representative examples of double-labelled phospho-tau- (p-Tau) and ROCK1-immunoreactive tangles at the different stages of AD indicated at left. Brain tissue samples from healthy aged controls (N = 5 aged 89 ± 4 y, Braak neuritic stages 0–2), preclinical AD (N = 4 aged 85 ± 3 y, Braak neuritic stages 3–4) and clinical end-stage AD (N = 8 aged 86 ± 4 y, Braak neuritic stages 5–6). All data are expressed as mean ± SD with at least three replications of the Western blot data. Scale in (**D**) is equivalent for all micrographs.

While changes in the levels of microRNA-146a and the phosphorylation of PTEN and tau are well established in brain regions targeted by AD (see above), changes in the levels of ROCK1 have not been evaluated. We therefore assessed the brain tissue levels of ROCK1 protein in the inferior temporal lobe of healthy aged controls (N = 5 aged 89 ± 4 y, Braak neuritic stages 0–2), preclinical AD (N = 4 aged 85 ± 3 y, Braak neuritic stages 3–4) and clinical end-stage AD (N = 8 aged 86 ± 4 y, Braak neuritic stages 5–6) using Western blotting of different tissue fractions (Fig. 3A and Supplementary Fig. 2). The soluble but not the sodium dodecyl sulfate (SDS) soluble protein levels were significantly reduced by 85% by end-stage AD (F_2_ = 4.9, *p* = 0.024, Fig. 3B) and negatively correlated with Braak neurofibrillary tangle staging (Rho = −0.502, *p* = 0.04). This is the first demonstration of a reduction in ROCK1 levels that is associated with AD pathology. Such a reduction would lead to reduced phosphorylation of PTEN in AD, as previously demonstrated^31^, and the reduction in PTEN phosphorylation would lead to a loss of tau dephosphorylation and to its hyperphosphorylation, as previously shown experimentally^27^ and consistent with the relationship to Braak neurofibrillary tangle staging shown in the present study.

To further identify whether ROCK1 was located in human pyramidal neurons, and particularly those containing hyperphosphorylated tau-immunoreactive neurofibrillary tangles, double labeling immunofluorescence was performed on formalin-fixed, paraffin-embedded tissue sections from the same cases. Quantitation of the overlap with hyperphosphorylated tau-immunoreactive neurofibrillary tangles revealed that ROCK1 colocalisation was present in an increasing number of phospho-tau-immunopositive tangles over the course of AD (Fig. 3C,D, F_2_ = 8.3, *p* = 0.004). The increase in ROCK1-immunoreactive neurofibrillary tangles correlated with increasing stage of AD pathology (Rho = 0.606, *p* = 0.022). Although fewer phospho-tau-immunoreative tangles were observed at the very earliest stages of AD, at these earlier stages all phospho-tau-immunoreactive tangles also colocalised ROCK1 immunoreactivity (Fig. 3C,D), indicating a close association of ROCK1 protein with early tangle formation, consistent with its binding to PTEN^29^,^30^,^36^. In fact, PTEN has a very similar localization in phospho-tau-immunopositive tangles in the same brain region in AD cases^38^, a location also demonstrated by many kinases known to phosphorylate tau^39^,^40^,^41^,^42^,^43^,^44^. This data is consistent with the concept that these proteins are important for tau phosphorylation and dephosphorylation, and that dysregulation of these intracellular systems associates with the characteristic intraneuronal pathology of AD. Of more interest though, is the association of RNA species with neurofibrillary tangles in AD^45^. While speculative, the RNA may include the microRNA-146a that is significantly increased early in tau-affected brain regions in AD^20^,^21^,^22^,^23^. Overall our data suggest that the increase in microRNA-146a previously detected in brain tissue vulnerable to AD would reduce neuronal ROCK1 protein levels, as we have now shown. Further, we show that the reduction in ROCK1 protein levels closely associates with increasing amounts of hyperphosphorylated tau pathology, as predicted from our neural culture experiments.

### Intra-hippocampal delivery of microRNA-146a inhibitor reduces tau hyperphosphorylation and enhances memory function in 5xFAD mice

**Figure 4:**
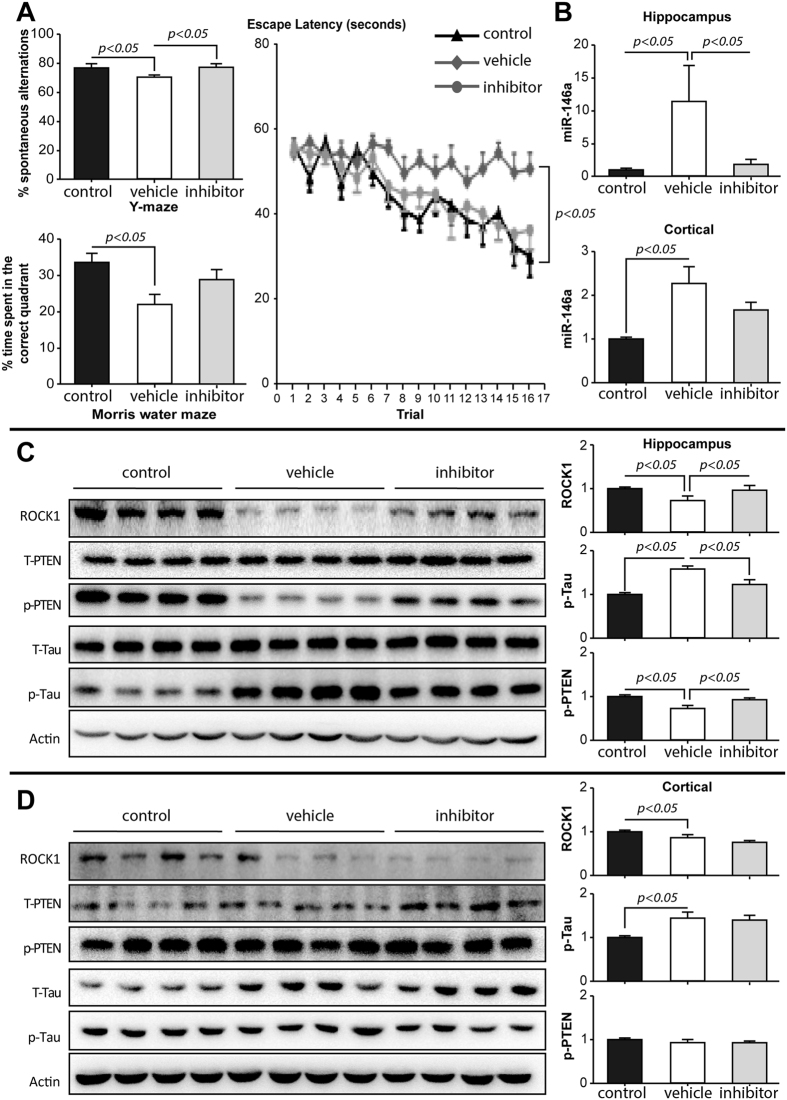
Intra-hippocampal delivery of antagomir. (**A**) The Y maze revealed that the percentage of spontaneous alternations was higher in 5xFAD mice treated with antagomir compared with Vehicle treated 5xFAD mice; and the Morris water maze showed shorter escape latency and higher percentage of time spent in the correct quadrant for 5xFAD mice treated with antagomir compared to Vehicle treated 5xFAD mice. (**B**) The relative expression of microRNA-146a (miR146a) was significantly inhibited in hippocampus, but not prefrontal cortex, after bilateral intra-hippocampal injections (*n* = 4). (**C**) The hippocampal protein levels of the related signaling pathway markers (ROCK1-pPTEN-pTau) were evaluated by Western blotting. A representative gel image is shown from four Control (wild-type), four Vehicle treated 5xFAD, and four 5xFAD with antagomir mice. (**D**) The prefrontal protein levels of the related signaling pathways (ROCK1-pPTEN-pTau) were evaluated by Western blotting. A representative gel image is shown from four Control (wild-type), four Vehicle treated 5xFAD, and four 5xFAD mice treated with antagomir mice. The data are shown as mean ± SE.

To determine whether reducing microRNA-146a could ameliorate the pathologies identified above, we assessed its levels and the use of a microRNA-146a inhibitor (antagomir) in a validated mouse model of AD, the 5xFAD mice^46^. The antagomir solvent control was delivered into the hippocampus of 5xFAD mice at three months of age and memory was tested in all mice including Control [wild-type mice], Vehicle [Sham treated 5xFAD mice] and Inhibitor [antagomir treated 5xFAD mice]) using the Y maze and Morris water maze before extracting brain samples for quantitative measurements of microRNA-146a and protein targets. Importantly, memory deficits were observed in conjunction with increased microRNA-146a levels in the Vehicle group compared to Control mice (Fig. 4A,B).

The 5xFAD mice treated with antagomir had a higher percentage of spontaneous alternations in the Y-maze, a higher percentage of time spent in the correct quadrant in Morris water maze and a shorter escape latency compared to Vehicle treated 5xFAD mice, indicating that antagomir increased hippocampal memory function (Fig. 4A). In parallel, the related signaling pathways (ROCK1-PTEN-Tau) were evaluated in the hippocampus and prefrontal cortex. Successful inhibition of microRNA-146a was achieved in the hippocampus following bilateral intrahippocampal injections of the microRNA-146a antagomir, while the levels of microRNA-146a in prefrontal cortex remained unchanged (Fig. 4B). The levels of ROCK1 protein and phospho-PTEN protein in the hippocampus but not prefrontal cortex were rescued in 5xFAD mice treated with intrahippocampal microRNA-146a antagomir compared to Vehicle treated 5xFAD mice, and a reduction in phospho-tau was observed (Fig. 4C,D). These hippocampal changes are consistent with the changes observed in our neural cell models. Thus, the improvement of memory by intrahippocampal microRNA-146a antagomir was associated with the predicted alterations in the ROCK1-PTEN-tau neural pathway, confirming that inhibition of microRNA-146a expression has a therapeutic effect in the 5xFAD transgenic mouse model of AD. This data support the concept that microRNA-146a antagomir is a potential efficacious therapeutic target for the tau pathology of AD.

## Conclusion

Inhibition of microRNA-146a expression with antagomir not only enhanced ROCK1 protein translation and PTEN phosphorylation, decreasing tau hyperphosphorylation, but also partly reversed cognitive impairment in 5xFAD mice. Our data strongly support the concept that in AD microRNA-146a upregulation contributes to tau hyperphosphorylation and cytoskeleton disruption by impeding the ROCK1-PTEN signaling pathway. While several microRNAs are known to promote the pathogenesis of AD by regulating tau phosphorylation, most of these microRNAs decrease within the brain as tau pathology occurs^47^, in contrast to the increase observed in microRNA-146a (see above). As microRNAs are repressors of protein translation, this data suggests opposing protein changes would occur in AD with these different microRNA changes - up-regulation of neural proteins associated with many other tau regulating microRNAs^47^ and down-regulation of neural proteins as observed for ROCK1 in the neural cells examined in the present study. MicroRNA antagomir treatment for tau hyperphosphorylation would only be successful for the increased microRNA-146a and not those microRNAs shown to be decreased with tau hyperphosphorylation^47^.

**Figure 5:**
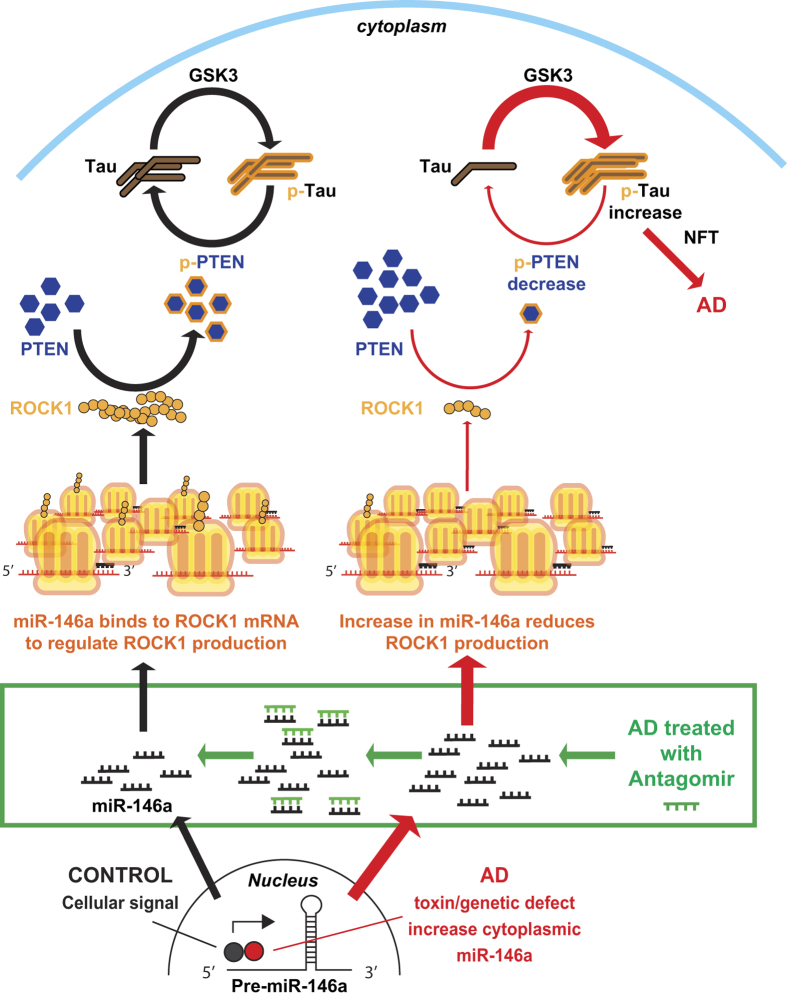
A model of the proposed inductive effects of microRNA-146a in Alzheimer’s disease (AD). Normal cell signaling (such as by NF-κB) stimulates the generation of the primary microRNA-146a (miR-146a) transcript that is produced in the nucleus aspre-microRNA-146a (Pre-miR-146a), which is then shuttled into the cytoplasm and further processed. MiR-146a binds to complimentary sequences in the 3′ untranslated region (UTR) of its target mRNA transcript of ROCK1. This results in translational repression of ROCK1 protein. Tau phosphorylation (p-Tau) and dephosphorylation are maintained in a dynamic balance for normal cell functions. Protein kinases (such as GSK3 etc) phosphorylate tau, while phosphorylated PTEN (p-PTEN) dephosphorylates tau. The levels of p-PTEN are regulated by the levels of ROCK1 protein, indicating that ROCK1 plays an important role in tau phosphorylation. In AD (red pathway at right) neuronal cells produce more miR-146a that decreases the levels of ROCK1 protein and reduces the levels of p-PTEN, impeding tau dephosphorylation. Thus, p-tau accumulates in neurons to form neurofibrillary tangles (NFT), finally leadingto neuronal death in AD. Treatment with antagomir neutralizes miR-146a (green box) restoring ROCK1 protein levels and facilitating appropriate PTEN phosphorylation and tau dephosphorylation.

In conclusion, we propose the following model for the role of microRNA-146a in the pathogenesis of AD (Fig. 5). Dysregulated microRNA-146a that is expressed at higher than normal levels in neurons negatively regulates ROCK1 protein translation. The reduction in neuronal ROCK1 protein leads to reduced neuronal PTEN phosphorylation and subsequently causes impairment of neuronal tau dephosphorylation. The current proposed model explains how microRNA-146a-mediated abnormalities in ROCK1 protein translation could contribute to the pathogenesis of AD. Moreover, inhibition of microRNA-146a expression with antagomir not only reverses the protein levels and/or phosphorylation of crucial signaling pathway elements (ROCK1-PTEN-Tau) in the brain, but also partly attenuates associated cognitive impairment. In general, we conclude that dysregulation of microRNA-146a biogenesis contributes to AD pathogenesis, and inhibition of this microRNA could have an application as an *in vivo* therapeutic.

## Methods

### Design of the study

Cell culture studies using SH-SY5Y neural cells were used to assess whether ROCK1 is a target of microRNA-146a in neural cells. Manipulation of microRNA-146a and ROCK1 in the SH-SY5Y neural cells was performed to determine the consequences for PTEN and tau phosphorylation. To determine if ROCK1 was changed in AD and whether there was any association with tau hyperphsophorylation in neurofibrillary tangles human brain tissue samples were studied. The 5xFAD murine animal model of AD was used to determine if reducing microRNA-146a in the hippocampus could ameliorate clinical symptoms and reduce tau phosphorylation through changing the ROCK1-PTEN-Tau pathway. The ethics committees of Shanghai Jiaotong University School of Medicine, China and University of New South Wales, Sydney Australia approved all experimental protocols. In China, the methods were carried out in accordance with the Chinese code for the care and use of animals for scientific purpose, and in Australia in accordance with National statement on ethical conduct in human research. For antibodies and reagents, see Supplementary Information.

### Cell culture conditions and experiments

SH-SY5Ycells with and without the stable overexpression of the 695 amino acid isoform of the human amyloid precursor protein (APP) gene were cultured in Dulbecco’s modified eagle medium (DMEM) supplemented with 10% heat inactivated fetal bovine serum in a humidified atmosphere of 5% CO_2_ at 37 °C. All culture materials were purchased from Invitrogen. To produce microRNA-146a loaded viral vectors for transfection experiments into SH-SY5Y cells, retroviral transduction of microRNA-146a was performed in HEK293T cells, as detailed in the Supplementary Information. To generate SH-SY5Y cell lines that stably express micoRNA-146a or scrambled micoRNA-146a, cells were infected with the retroviral supernatants by spinoculation in 6-well plates, as detailed in the Supplementary Information.

Luciferase reporter assays were performed in SH-SY5Y cells. The full length 3′ UTR of human ROCK1 was digested with *Not*I and *Xho*I and then cloned into the psiCHECK-2 vector (Promega). The ROCK1 3′ UTR was PCR amplified from human genomic DNA using the following primers: forward: 5′-TCGCTCGAGCCATGTGACTGAGTGCCCTG-3′ and reverse: 5′-TCGGCGGCCGCTATCTTTTAAAAATACACTTT-3′. To construct a ROCK1 3′ UTR reporter lacking the potential binding site for microRNA-146a, the following primers were used: forward: 5′-TCGCTCGAGGAGGTTTGTTGGACTTTC-3′ and reverse: 5′-TCGGCGGCCGCTATCTTTTAAAAATACACTTT-3′. The constructs were confirmed by sequencing. SH-SY5Y cells were plated in 96-well plates at 5,000 cells per well the day before transfection. Transfection was performed in triplicate with Lipofectamine 2000 and 100 ng of plasmid consisting of 90 ng of either the microRNA-146a expression vector or the scrambled control vector and 10 ng of the reporter vector. The scrambled control sequences were sense 5′-UUCUCCGAACGUGUCACGUTT-3′ and antisense 5′-ACGUGACACGUUCGGAGAATT-3′. Small interfering ROCK1 RNA (siRNA, GenePharma, Shanghai) knockdown compared to scrambled ROCK1 siRNA was also used as experimental controls. The scrambled ROCK1 siRNA sequences were sense 5′-UUCUCCGAACGUGUCACGUTT-3′ and antisense 5′-ACGUGACACGUUCGGAGAATT-3′. Transfections were performed according to the manufacturer’s protocol using Lipofectamine 2000 combined with 100 nM ROCK1 or scrambled ROCK1 siRNA. Forty-eight hours post-transfection, luciferase activity was assayed on a Micro-plate Spectrophotometer (BIO-TEK Instruments, USA) using the Dual-Luciferase^®^ Reporter Assay System according to the manufacturer’s protocol (Promega). The renilla luciferase activity was used to normalize to the firefly luciferase activity in each well.

Cells were harvested for both mRNA analysis and protein analysis. Total RNA was isolated using Trizol reagent (Invitrogen, China). Proteins were extracted using RIPA buffer (50 mM TrisHCl pH 8.0, 150 mM NaCl, 1% NP-40, 0.5% sodium deoxycholate, and 0.1% SDS) with a protease inhibitor cocktail (Roche) and 30 μg of protein loaded onto a 10% SDS–PAGE gel for Western blotting.

### Pathological cohort

The human ethics committee of the University of New South Wales approved this tissue study. Following institutional approvals, brain tissue (500 mg fresh temporal cortex and 10 μm formalin-fixed paraffin-embedded temporal cortex sections) from 17 cases with different severities of AD pathology (female: male = 13:4, age = 87 ± 9.5 (years old), post mortem delay = 15 ± 11 (hours), low severity, Braak stages 0–2 and not demented: intermediate severity and Braak stages 3–4: high severity, Braak stages 5–6 and dementia = 5:3:9) were obtained from the Sydney Brain Bank which collects brain tissue with informed consent from longitudinally followed research participants. Subjects were characterised according to recent criteria for the pathological diagnosis of AD^48^,^49^. One of the cases with an intermediate severity had had a diagnosis of AD for three years, while all those with a high probability of AD had dementia for between five and 17 years. Exclusion criteria were alternate neurodegenerative disorders or a dominant family history of neurodegenerative disorder.

Tissue fractionation was performed with 500 mg of fresh brain tissue dissected from temporal lobe of each subject and homogenised using a Potter-Elvehjem apparatus fitted with a Teflon pestle in three ml homogenisation buffer (0.32 M sucrose, 20 mM Tris, 5 mM ethylenediaminetetraacetic acid or EDTA, containing proteinase inhibitors). The homogenates were sonicated at 2 × 10 seconds on ice and the solution centrifuged at 120,000 g for one hour at 4 °C to obtain the supernatant or soluble fraction, and then the pellet was washed with homogenisation buffer and centrifuged at the same speed for 10 minutes. The supernatant was discarded and the pellets were collected, solubilised in the homogenisation buffer containing 5% SDS. The SDS solution was further centrifuged at 100,000 g for half an hour at 25 °C. The supernatant were collected as the SDS fraction, and the pellets were solubilised in 8% SDS/8 M urea solution. Protein concentration was measured using a BCA kit (Thermo scientific), and 30 μg of total protein from each sample loaded onto a 4–15% Criteron gel with 18 wells (Bio-Rad) for Western blotting.

### Experiments using a transgenic mouse model of Alzheimer’s disease (AD)

The Institutional Animal Care and Use Committee of Shanghai Jiaotong University School of Medicine approved this animal study. We used 5xFAD mice^46^ (Model Animal Research Center of Nanjing University, Nanjing, China) that harbor three mutations, two in the presenilin-1 gene and the APP695, and accumulate soluble Aβ_1–42_ and develop plaques by four to five weeks of age. Mice were generated by crossing heterozygous 5xFAD mice to heterozygous 5xFAD mice in C57BL/6 background mice. Wild-type C57BL/6 littermate control mice served as age-matched control animals.

Mice aged 17 weeks were anesthetized with chloral hydrate (300 mg/kg) intraperitoneally. The anesthetized mice were placed in a stereotactic apparatus for intra-hippocampal injections at coordinates in mm relative to bregma of AP: −2.0, L: 1.8, DV: −1.5. Bilateral injections of one μL (0.5 nmol) micoRNA-146a inhibitor or solvent were performed using a 10 μL Hamilton syringe at a rate of 0.2 μL/min. Five minutes after the completion of the injection, the needle was slowly withdrawn from the animal. After sufficient awakening from anesthesia, animals were returned to their cages for long-term recovery.

Tests were performed using Y maze and the water maze (see Supplementary Information for details). Both sexes of 5xFAD mice and wild-type controls were tested (n = 13 mice with 6 male and 7 female for each 5xFAD group, and n = 15 mice with 7 male and 8 female for each wild-type group).

### Preparation of mice brain extracts for protein analysis

One day after the end of behavioral tests, anesthetized mice were decapitated and brains immediately extracted. Homogenates of hippocampus and cortex were prepared in extraction buffer [RIPA, 50 mmol/L TrisHCl (pH 8.0), 150 mmol/L NaCl, 1% NP-40, 0.5% sodium deoxycholate, and 0.1% SDS] with protease inhibitor cocktail and p-cocktail (Roche). After 30 minutes incubation on ice, the homogenates were then centrifuged at 12,000 g for 30 minutes at 4 °C, and the supernatant taken for further analysis.

### Real-time quantitative PCR for RNA analyses

Real-time quantitative PCR for microRNAs was performed using the SYBR PrimeScript™ microRNA RT-PCR Kit (Takara, China). Briefly, 5 μg of RNA was converted into cDNA. cDNA was synthesized with PrimeScript 1st Strand cDNA Synthesis Kit (Takara, Japan). Real-time quantitative PCR was performed with an ABI prism 7500 system. The micoRNAs assessed included hsa-microRNA-146a and pre-hsa-microRNA-146a. The PCR primers used were: hsa-microRNA-146a: 5′-GCTGAGAACTGAATTCCATGGGTT-3′. U6 was used as the control. The U6 forward and reverse primers were 5′-CTCGCTTCGGCAGCACA-3′ and 5′-AACGCTTCACGAATTTGCGT-3′. To assess ROCK1 mRNA expression, the SYBR^®^ Premix Ex Taq^TM^ Kit was used (Takara, China). The forward and reverse primer sequences for ROCK1 mRNA amplification were 5′-TGAGCAACTATGATGTGCCTGAA-3′ and 5′-AGCGTTTCCCAAGCCCACT-3′. The relative expression of ROCK1 mRNA was normalized to glyceraldehyde 3-phosphate dehydrogenase (GAPDH). The GAPDH forward and reverse primers were 5′-CGGAGTCAACGGATTTGGTCGTAT-3′ and 5′-AGCCTTCTCCATGGTGGTGAAGAC-3′ respectively. The expression of target genes was measured using the 2^−ΔΔCt^ method^50^. All of the reactions were performed in triplicate.

### Western blotting

Protein gels were transferred to nitrocellulose membranes (human) or polyvinylidenedifluoride membranes (cells) and blocked with skim milk (human) or 5% bovine serum albumin (cells), then incubated with the primary antibody prior to secondary antibody detection. The intensities of the protein bands were captured using a Chemidoc BP (Bio-Rad) and quantified using Image Lab Software (Bio-Rad) normalized to β actin (human) or GAPDH (cell) expression. Each experiment was repeated more than twice and the data averaged.

### Immunofluorescence of transfected cells and human brain tissue sections

Transfected cells were washed twice in ice-cold phosphate-buffered saline (PBS), and then fixed in 4% paraformaldehyde in PBS for 15 minutes at room temperature. The fixed cells were permeabilised in 0.1% Triton X-100 in PBS for 5 minutes. After three washes with PBS, the cells were incubated in blocking solution (PBS containing 5% BSA), for one hour to reduce non-specific binding. Next, the cells were incubated with primary antibodies in blocking solution for two hours, washed three times in PBS, and then incubated for one hour with the linked secondary antibodies. The cells were then washed three times with PBS. Coverslips were mounted using Prolong Gold anti-fading reagent with 4′,6-diamidino-2-phenylindole (DAPI, Invitrogen). Immunofluorescence was captured using a phase contrast fluorescence microscope (Nikon Eclipse E400).

Ten μm formalin-fixed paraffin-embedded sections of the inferior temporal cortex were used for ROCK1 and phospho-tau immunohistochemistry using both horseradish peroxidase (for light microscopy) and double immunofluorescence (for confocal microscopy). Quantitation of the number of phospho-tau-immunoreactive structures colocalising ROCK1 immunostaining was performed from ten representative images of the cortex that contained phospho-tau immunoreactive tangles for each case at X 600 magnification using a Nikon microscope C1 (EZ-C1, Nikon Instruments, Melville, NY, USA) and the numbers expressed as the total numbers observed in the samples per case. A total of 460 phospho-tau immunoreactive tangles were sampled in the ten images per case (ranged from 0–104 phospho-tau immunoreactive tangles assessed/case). Due to case selection for minimal cortical pathology in non-AD cases, only ~50 of the 460 phospho-tau immunoreactive tangles were found in the cortices of the non-AD cases with three cases having no tangles as required for Braak stages of 0–2.

### Statistical analyses

Analyses were performed using SPSS software (IBM SPSS statistics 21) with a *p*-value of ≤0.05 considered as significant. Normality of the data was tested using Shapiro-Wilk test. Non-parametric data were transformed using Rank cases by Blom’s Formula prior to analysis, and normal distributions confirmed by histograms. A single multivariate statistical analysis between groups stratified by the severity of AD pathology was used to assess differences in the levels of TBS-soluble and SDS-soluble ROCK1 protein levels and the comparative numbers of sampled phospho-tau-immunoreactive tangles that colocalised ROCK1 immunoreactivity, covarying for age and post-mortem delay. Correlations between variables were tested using Spearman Rho analysis. Categorical variables were compared using the χ^2^ test.

## Additional Information

**How to cite this article**: Wang, G. *et al*. MicroRNA-146a suppresses ROCK1 allowing hyperphosphorylation of tau in Alzheimer’s disease. *Sci. Rep.* **6**, 26697; doi: 10.1038/srep26697 (2016).

## Supplementary Material

Supplementary Information
