# Supplementary material for: MicroRNA-146a suppresses ROCK1 allowing hyperphosphorylation of tau in Alzheimer’s disease
Source: Sci Rep. 2016 May 25;6:26697. doi: 10.1038/srep26697 (PMC4879631; doi:10.1038/srep26697)
Supplement: Supplementary Information [file srep26697-s1.pdf]

Supplementary Information

Title: MicroRNA-146a suppresses ROCK1 allowing hyperphosphorylation of tau in Alzheimer's disease

Gang Wang[1*] MD&PhD, Yue Huang[2*] MD&PhD, Li-Ling Wang[1*] PhD, Yong-Fang Zhang[3*] PhD, Jing Xu[1] MD, Yi Zhou[1] MD, Guinevere F. Lourenco[2] BS&MBiotech, Bei Zhang[1] MD, Ying Wang[1], Ru-Jing Ren[1] MD&PhD, Glenda M. Halliday[2#] PhD, Sheng-Di Chen[1,4,#] MD&PhD

[*]These four authors (GW, YH, LLW, YFZ) contributed equally to this work.

* These four authors (GW, YH, LLW, YFZ) contributed equally to this work.

[1]Department of Neurology & Neuroscience Institute, Ruijin Hospital affiliated to Shanghai Jiao Tong University School of Medicine, Shanghai, 200025, China; [2]Neuroscience Research Australia & University of New South Wales, Randwick, 2031, New South Wales, Australia; [3]Research Laboratory of Cell Regulation, School of Medicine, Shanghai Jiaotong University, 280 South Chongqing Road, Shanghai 200025, China; [4]Laboratory of Neurodegenerative Diseases, Institute of Health Science, Shanghai Institutes of Biological Sciences, Chinese Academy of Sciences and Shanghai Jiao Tong University School of Medicine, Shanghai 200025, China

Supplementary Figure 1 - The predicted conserved microRNA-146a binding sites within the ROCK1 3′ UTR are shown blocked and underlined, and the motif used for the truncated ROCK1 3′ UTR is highlighted in grey.

>gi|112382209|ref|NM_005406.2| Homo sapiens ROCK1|3′UTR

TAACCATGTGACTGAGTGCCCTGTGGAATCGTGTGGG
ATGCTACCTGATAAACCAGGCTTCTTTAACCATGCAGAGCAGACAGGCTGTTTCTTTGACACAAATATCA
CAGGCTTCAGGGTTAAGATTGCTGTTTTTCTGTCCTTGCTTTGGCACAACACACTGAGGGTTTTTTTTAT
TGCGGGTTTGCCTACAGGTAGATTAGATTAATTATTACTATGTAATGCAAGTACAGTTGGGGGAAAGCTT
AGGTAGATATATTTTTTTTTAAAAGGTGCTGCCTTTTTGGATTTATAAGAAAATGCCTGTCAG**TCGTGATA**
GAACAGAGTTTTCCTCATATGAGTAAGAGGAAGGGACTTTCACTTTCAAGTGGAACAGCCATCACTATCA
AGATCAGCTCATGGAAGGAGTAAAGAAAATATCTCAAAATGAGACAAACTGAAGTTTTGTTTTTTTTTA
ATGACTTAAGTTTTTGTGCTCTTGCAAGACTATACAAAACTATTTTAAGAAAGCAGTGATATCACTTGAA
CTTCAGTGCCCTCACTGTAGAATTTAAAAGCCTTACTGTTGATTGCCCATGTTGGACTTGATGGAGAAAT
TAAATATCTTTCATTATGCTTTACAAAATACTGTATATGTTTCAGCAAGTTTGGGGAATGGGAGAGGACA
AAAAAAAGTTACATTTAATCTATGCATTTTTGCCAAGCCATATTGAGTTATTTTACTACTAGAGACATTA
GGAAACTAACTGTACAAAAGAACCAAGTTTAAAAGC**ATTTTGTGGGGTACATCATTTCTAT**AATTGTATA
ATGTATTTCTTTGTGGTTTTAAATGATAAAGACATTAAGTTAACAAACATATAAGAAATGTATGCACTGT
TTGAAATGTAAATTATTCTTAGAACACTTTCAATGGGGGTTGCATTGTCCTTTTAGTGCCTTAATTTGAG
ATAATTATTTTACTGCCATGAGTAAGTATAGAAATTTCAAAAAATGTATTTTCAAAAAAT**TATGTGTGTC**
AGTGAGTTTTTCATTGATAATTGGTTTAATTTAAAATATTTAGAGGTTTGTTGGACTTTCATAAATTGAG
TACAATCTTTGCATCAAACTACCTGCTACAATAATGACTTTATAAAACTGCAAAAAATGTAGAAGGTTGC
ACCAACATAAAAAGGAAATATGGCAATACATCCATGATGTTTTCCAGTTAACATAGGAATTACCAGATAA
ATACTGTTAAACTCTTGTCCAGTAACAAGAGTTGATTCATATGGACAGTATGATTTATTGTTTATTTTTT
TAACCAAATACCTCCTCAGTAATTTATAATGGCTTTGCAGTAATGTGTATCAGATAAGAAGCACTGGAAA
ACCGATCGTCTCTAGGATGATATGCATGTTTCAAGTGGTATTGAAAGCCGCACTGATGGATATGTAATAA
TAAACATATCTGTTATTAATATACTAATGACTCTGTGCTCATTTAATGAGAAATAAAAGTAATTTATGGA
TGGGTATCTTTAATTTTTACTGCAATGTGTTTTCTCATGGCTGAAATGAATGGAAAACATACTTCAAATT
AGTCTCTGATTGTATATAAATGTTTGTGAAATTCCATGGTTAGATTAAAGTGTATTTTTAAAAGATAAAA

Supplementary Figure 2 – Full Western blots and replicate of ROCK1 protein levels in the different tissue fractions (TBS=soluble fraction, SDS=SDS fraction, see above) of the temporal lobe of donors at different stages of Alzheimer's pathology (B1=healthy aged controls, B2=preclinical AD, B3=clinical end-stage AD). The SDS soluble fraction revealed a second smaller molecular weight band that was not quantified.

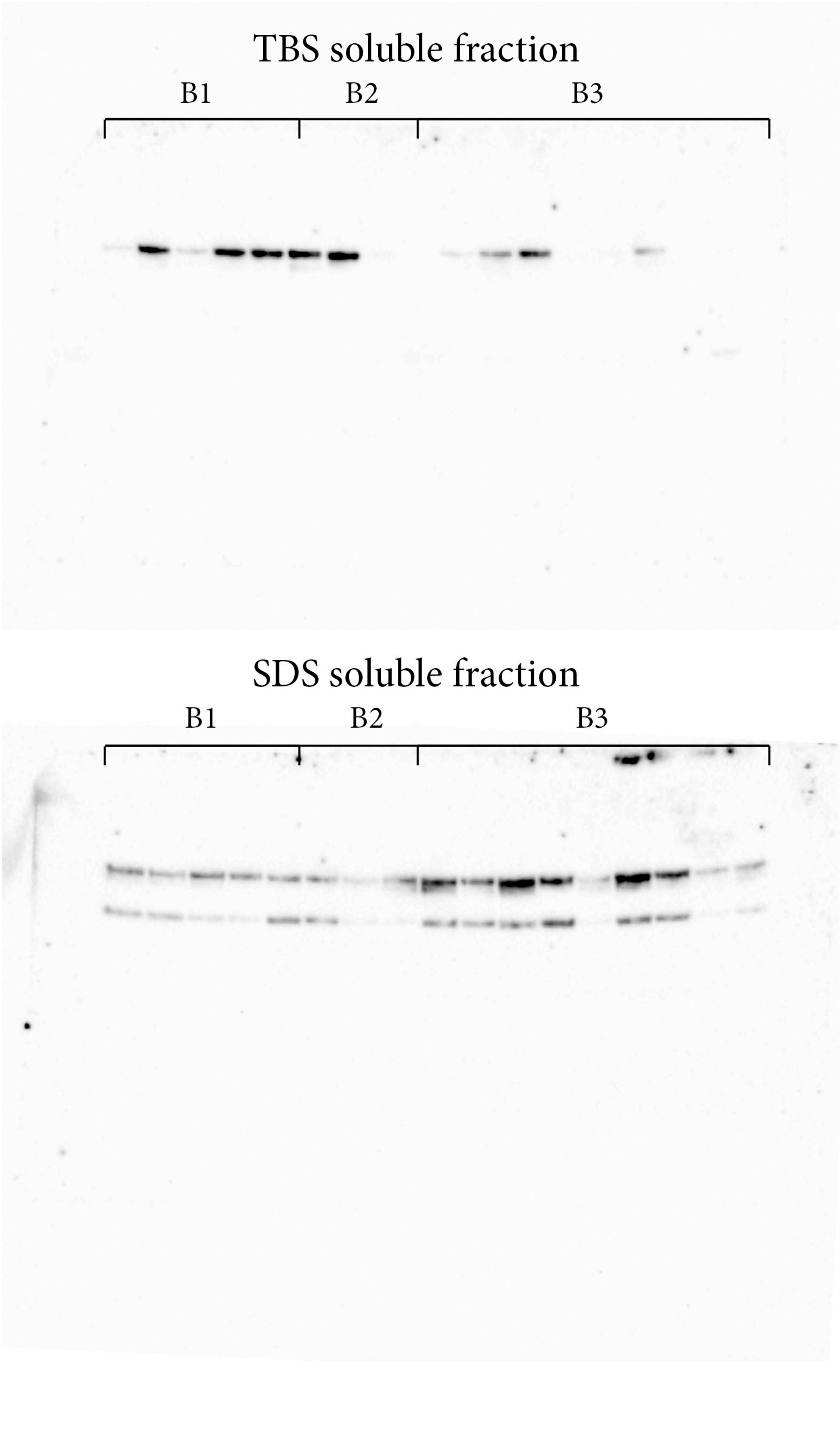

Replicate Western blots pasted below each fraction

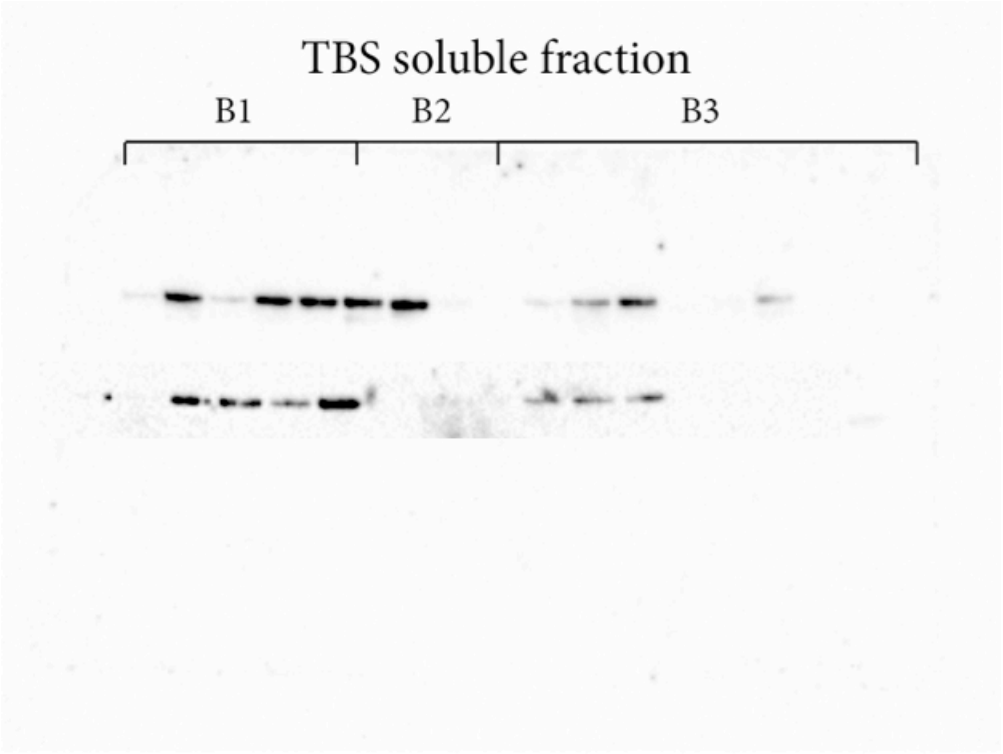

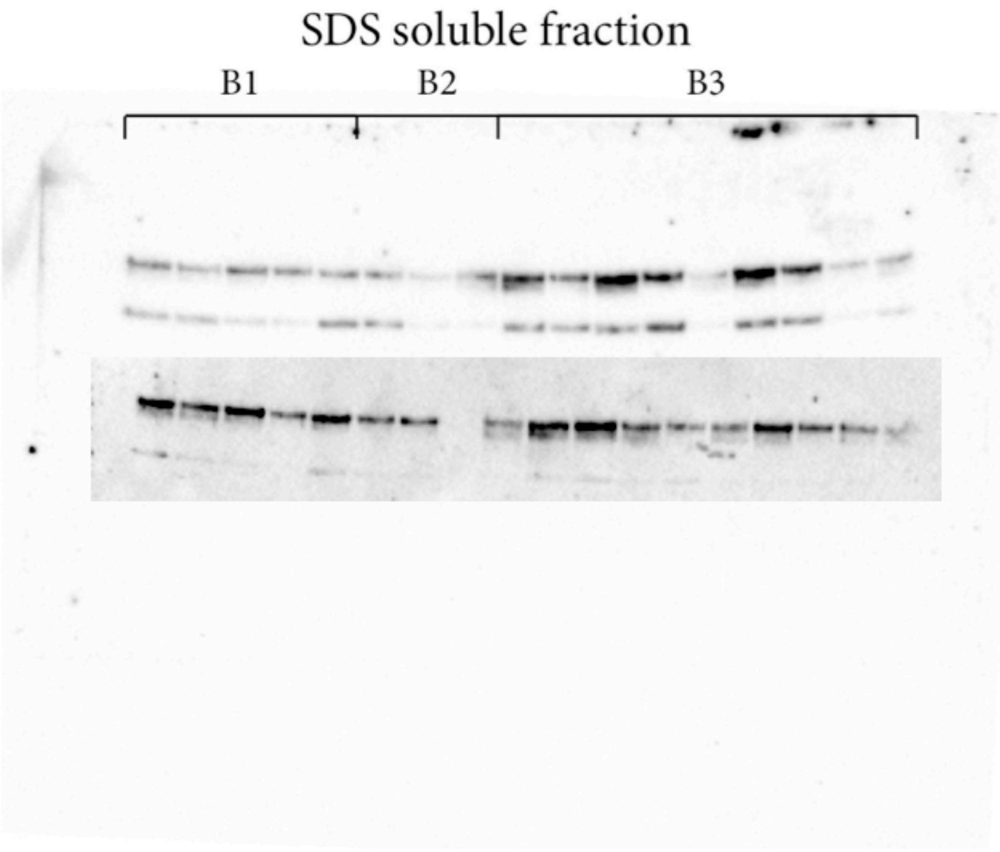

Production of microRNA-146a loaded and scramble loaded viral vectors

To produce microRNA-146a loaded viral vectors for transfection experiments into SH-SY5Y cells, retroviral transduction of microRNA-146a was performed in HEK293T cells. All culture materials were purchased from Invitrogen. The hsa-microRNA-146a was PCR amplified from human genomic DNA (Roche Applied science, USA) using the following primers: forward: 5′-AGATCTGGTCTCCTCCAGATGTTT-3′ and reverse: 5′-CTCGAGAGCTACTTGGAACCCTGCT-3′. The *Bgl* II and *Xho* I recognition sites in each primer are underlined. The product and a scrambled microRNA-146a sequence (GenePharma, Shanghai) were both cloned into modified MSCV-PGK-GFP vectors (Clontech). Viral vectors were made by transfecting HEK293T cells using Lipofectamine 2000 (Invitrogen) according to the manufacturer's instructions. Briefly, 10 μg of expression vectors or empty vector with 10 μg packing plasmid (gag/pol and VSVG) were incubated with Lipofectamine 2000 for 20 minutes at room temperature and added to HEK293T cells. The viral supernatant was harvested after 48 hours.

Generation of SH-SY5Y cells that stably express microRNA-146a or scrambled microRNA-146a

To identify whether ROCK1 was a target of microRNA-146a in neuronal cells, SH-SY5Y cells were cultured in Dulbecco’s modified eagle medium (DMEM) supplemented with 10% heat inactivated fetal bovine serum in a humidified atmosphere of 5% CO_2 at 37°C. All culture materials were purchased from Invitrogen. MicroRNA-146a loaded or scramble loaded viral vectors, produced as detailed above, were used to infect cells by spinoculation in 6-well plates. Successful transfection of SH-SY5Y cells was confirmed by a significant increase in microRNA-146a (**Supplementary Table 1 and Supplementary Figure 3**).

Supplementary Table 1. rt-PCR measurements of microRNA-146a in control and transfected SHSY-5Y cells

Cells	N	Mean±SD	SEM	95% confidence interval
SHSY-5Y controls	3	1.00±0.00	0.00	1.00-1.00
SHSY-5Y with miRNA-146a scramble	3	3.38±1.94	1.12	-1.45-8.21
SHSY-5Y with miRNA-146a	3	32.79±1.73*	1.00	28.51-37.08
Total	9	12.39±15.39	5.15	0.56-24.22

*significantly increased from control cells using t test p<0.05

Supplementary Figure 3. Successful transfections of microRNA-146a into SHSY-5Y cells demonstrated by the mean difference in microRNA-146a mRNA levels

RT-PCR confirmed successful transfer of miRNA146a in SH-SY5Y

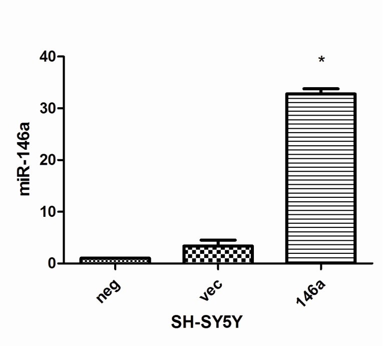

	Group (I)	Group (J)	Mean differences (I-J)	SD	p
LSD	Neg	Scramble	-2.38	1.23	0.10
		miRNA-146a	-31.79	1.23	0.00
	Scramble	Neg	2.38	1.23	0.10
		miRNA-146a	-29.41	1.23	0.00
	miRNA-146a	Neg	31.79	1.23	0.00
		Scramble	29.41	1.23	0.00

Neg = SH-SY5Y
Vec = SH-SY5Y transferred with scramble miRNA146a
146a = SH-SY5Y transferred with miRNA146a

Y maze test for transgenic mouse model of Alzheimer's disease

A black Y-maze made of steel with each arm 45 cm long, 10 cm wide and 15 cm high, and the three arms positioned at equal angles was used. Each mouse was placed in an arm facing the center (Arm A) and was allowed to explore freely through the maze for 5 minutes. A spontaneous alternation occurred when the animal moved to the other two arms without retracing its steps (i.e. Arm A to B to C). Movements such as ABA were incorrect. Based on the movement over the entire session, the percentage of spontaneous alternations was calculated.

Water maze test for transgenic mouse model of Alzheimer's disease

The water maze consisted of a large black circular pool (120 cm diameter), filled with water. The pool was divided into four equal quadrants, and a black platform (9 cm diameter) submerged one cm below the surface of the water in the center of one quadrant (target quadrant). The platform was made invisible to the mice and remained in one location for the entire test. A high-resolution camera was suspended over the center of the pool, its images being monitored by a video-tracking system (Morris Water Maze Video Analysis System [DigBeh-MM], Shanghai Jiliang Software Technology Co. Ltd, Shanghai, China). The experiment began with one day of cued training, during which the platform was visible. Following this, each mouse performed four days of acquisition training. For this part of the testing, the platform was hidden. The daily order of entry into individual quadrants was randomized so that all four quadrants were used once every day. Each mouse received four training trials per day with an average inter-trial interval of 15 minutes. Escape latency was defined as the time required by the mouse to find the platform and climb onto it. The mouse was allowed to swim freely to seek the hidden platform for 60 seconds and was left for an additional 20 seconds on the platform. Latency was recorded for up to 60 seconds. If the mouse did not find the platform within 60 seconds, it was placed gently on the platform for 20 seconds. On day 6, the platform was removed and a 60 second probe trial was conducted twice. For all trials, peripheral cues around the maze environs remained constant throughout testing.
